# Evaluating the Effectiveness of Gentamicin-Impregnated Collagen Sponge in the Prevention of Surgical Site Infection in Colorectal Surgery: A Systematic Literature Review and Meta-Analysis

**DOI:** 10.7759/cureus.95798

**Published:** 2025-10-31

**Authors:** Andrew C Ekwesianya, Abraham Jesudoss, Abraham A Ayantunde

**Affiliations:** 1 Department of General and Colorectal Surgery, Southend University Hospital, Southend, GBR; 2 Department of General Surgery, Southend University Hospital, Southend, GBR

**Keywords:** chronic pilonidal disease, collagen fibres, lower gi or colorectal surgery, surgical site infection (ssi), surgical wound infection

## Abstract

Surgical site infection (SSI) is a frequent postoperative complication, particularly in colorectal surgeries, which exhibit higher SSI rates than other procedures. The application of Gentamicin-Collagen Implant (GCI) at a local surgical site maximises not only the unique qualities of collagen in enhancing wound healing but also the antibacterial effects of gentamicin. The objective of this study is to determine if SSI is reduced by the use of GCI during colorectal procedures. Included studies were randomised controlled trials (RCTs) comparing GCI use with a defined control in colorectal surgeries. Exclusions comprised non-RCTs, case reports, animal studies, non-colorectal surgery comparisons, and studies lacking relevant endpoints. Literature searches were conducted in January 2025 using the SCOPUS, PubMed, Embase, and Cochrane Library medical databases. Risk of bias was assessed with the Rob2 tool (Cochrane Collaboration, Oxford, UK), and a meta-analysis was conducted using Review Manager (Cochrane Collaboration). Endpoints included incisional SSI, primary wound healing, and disease recurrence. Fourteen RCTs with 2,233 patients (1,114 with and 1,119 without GCI) were analysed. GCI significantly lowered overall SSI incidence (RR = 0.54; CI = 0.35-0.85; p = 0.007) and specifically in rectal surgeries (RR = 0.56; CI = 0.39-0.81; p = 0.002). No significant effect was observed in pilonidal disease surgeries (RR = 0.34; CI = 0.09-1.23; p = 0.10) or its recurrence (RR = 0.62; CI = 0.09-4.57; p = 0.64). GCI effectively reduces SSI in rectal surgeries but not in pilonidal disease operations. Routine GCI may reduce SSI in rectal surgery.

## Introduction and background

Surgical site infection (SSI) is a common cause of morbidity and sometimes mortality in the early postoperative period [1]. Colorectal surgeries have a higher rate of SSI, compared to surgeries in other parts of the body, due to the higher count of bacteria per gram of bowel content and the effect of preoperative treatments, such as neoadjuvant radiotherapy, on the bowel [2]. Following abdominoperineal resection, wound infection rates have been reported to be as high as 60% [3], due to high ligation of the supplying arteries (for oncological benefits), large surface of the sacral wound, negative effects of neoadjuvant chemoradiotherapy on tissues and wound healing, and the bony boundaries of the pelvis; as a result, systemically administered antibiotics do not have effective concentrations in this area.

Many measures have been instituted to reduce the risk of SSI, including the use of systemic prophylactic antibiotics at the time of induction of anaesthesia, negative pressure wound devices, and open wound management for contaminated and dirty wounds. In colorectal surgery, antibiotics are often given preoperatively during mechanical bowel preparation to reduce transmural translocation of bacteria, a practice that has proven to reduce the incidence of SSI [4].

Current strategies to prevent surgical site infections (SSIs) in colorectal surgery are multifaceted. They include adherence to Enhanced Recovery After Surgery (ERAS) protocols, preoperative bowel preparation with oral antibiotics, and the use of negative-pressure wound therapy devices. Furthermore, the increasing adoption of minimally invasive techniques, such as laparoscopic and robotic surgery, has significantly influenced SSI risk by enabling smaller incisions, more precise tissue dissection, reduced blood loss, and decreased tissue trauma.

The use of drug-delivery systems to concentrate antibiotics at the local site of surgery to prevent SSI has been in practice for decades. This method is being used extensively in the management of various orthopaedic surgical procedures by vascular surgeons to prevent graft infections and to prevent sternal wound infections following cardiac surgeries [5-7]. There are also literature reports of the use of this system in colorectal surgical procedures, especially in low rectal surgeries, to prevent surgical site infections [8].

In contrast to the traditional synthetic antimicrobial delivering beads, which require a repeat surgery to explant and can actually promote wound infection through the formation of bacterial biofilm around the drug delivery device, using collagen as a delivery system has several proven advantages: it is a biological material that dissolves in tissues, promotes haemostasis and enhances tissue gluing at the site of surgery [9]. In addition, the porosity of the collagen matrix allows for rapid and sustained release of the impregnated antibacterial over a period of time [10].

Gentamicin is the choice agent for use in the collagen-antibacterial complex at the local site of surgery and appears in various forms such as Collatamp® (EUSA Pharma Oxford, United Kingdom) and Septocoll® (Biomet Deutschland GmbH, Berlin, Germany). Aerobic gram-negative bacilli and staphylococci are the predominant organisms responsible for surgical site infection following lower gastrointestinal and perineal surgeries, and these are sensitive to aminoglycosides such as gentamicin. Gentamicin withstands the industrial-level gamma ray sterilisation of collagen without losing its potency. In addition, gentamicin exhibits concentration-dependent killing and post-antibiotic effect, a phenomenon in which inhibition of bacterial growth continues even after the level of the antibiotic has fallen below the minimum inhibitory concentration [10].

Gentamicin-containing collagen sponges deliver a high concentration (above the minimal inhibitory concentration) of the antibacterial within the local surgical site, thereby killing bacteria that are known to be gentamicin-resistant [11]. Slow release of the drug for a longer period in the surgical site reduces systemic absorption and its attendant toxicity, decreases the risk of antibacterial drug resistance and the need for repeated systemic antibiotic administration [10].

There has not been any previous meta-analysis that has assessed the usefulness of Gentamicin-Collagen Implant (GCI) in preventing SSI in rectal surgery. The aim of this study, therefore, was to search available literature, collate relevant data and determine, through a meta-analysis, if SSI is reduced by the use of GCI during colorectal surgeries.

## Review

Eligibility criteria

The studies included in this review are randomised controlled trials (RCTs) that compared the use of GCI against a well-defined control during colorectal surgeries. The wound sites are the abdominal wound or the perineal wound, depending on the type of surgery.

The exclusion criteria were case reports or case series, animal studies, non-RCTs, comparative studies outside the realm of colorectal surgery, and studies that did not evaluate the desired endpoints.

Information sources and search strategy

The literature review was carried out in accordance with the PICO (Problem, Intervention, Comparison, Outcome) framework [12]. The literature search was performed in January 2025 on SCOPUS, PubMed, Embase and the Cochrane Library databases. The search terms used were ‘gentamicin’, ‘collagen’, ‘implant’, ‘surgical site infection’, ‘wound infection’, ‘colorectal’, ‘gentamicin-impregnated’, ‘collagen sponge’, ‘collagen fleece’, and ‘Collatamp’. Boolean operators (‘AND’, ‘OR’, ‘NOT’) were used to narrow or widen the search as appropriate. No sample size or language restrictions were imposed during the literature selection process.

Selection and data collection process

A preliminary screening of the search results was performed based on the title to exclude those with no relationship to the research topic. Subsequently, abstracts of the remnant articles were reviewed to exclude case series, animal studies and articles dealing with antibiotic implants in other body systems. The remaining articles were read in full, and relevant data were extracted from studies that had the desired outcome measures. References of the selected articles were also searched, and additional studies that met the inclusion criteria were identified and reviewed. The literature search and data selection were independently performed by two of the authors (ACE, AVJ) using a drafted template. Any discrepancy was resolved by discussion.

Study risk of bias and certainty of evidence assessments

The risk of bias assessment of the individual studies was performed using the Rob2 risk of bias assessment tool (Cochrane Collaboration, Oxford, UK), and presented along with a forest plot [13]. Certainty of evidence was assessed using the GRADEpro GDT (Guideline Development Tool) software [14].

Effect measures

The primary outcome of interest for this review was incisional surgical site infection (either superficial or deep), based on the criteria defined by the Centres for Disease Control and Prevention [15]. The secondary endpoints were the rate of primary wound healing and disease recurrence. In addition, other relevant study parameters were extracted, including the population within which the study was carried out, year of publication, type of study, study design, anatomical location of the wound, mean duration of post-operative follow-up, surgical pathology and nature of surgery performed.

Synthesis method

The systematic literature review was conducted in accordance with the Preferred Reporting Items for Systematic Reviews and Meta-Analysis (PRISMA) 2020 framework [16]. Data analysis was carried out using Cochrane’s meta-analysis software - Review Manager version 5.4 (The Cochrane Collaboration, Oxford, UK) [17]. Using the random effect model, the pooled risk ratio (RR) at 95% Confidence interval (CI) was calculated to determine the statistical difference in outcome between the treatment and control groups. Heterogeneity was assessed using the chi-squared test, with statistical significance set at a p-value of <0.05. The heterogeneity (I^2^) test was used to quantify the degree of heterogeneity, with I^2^ values <25% indicating low heterogeneity, 25-50% indicating moderate heterogeneity, and >50% signifying severe heterogeneity [18].

Results

Study Selection

**Figure 1:**
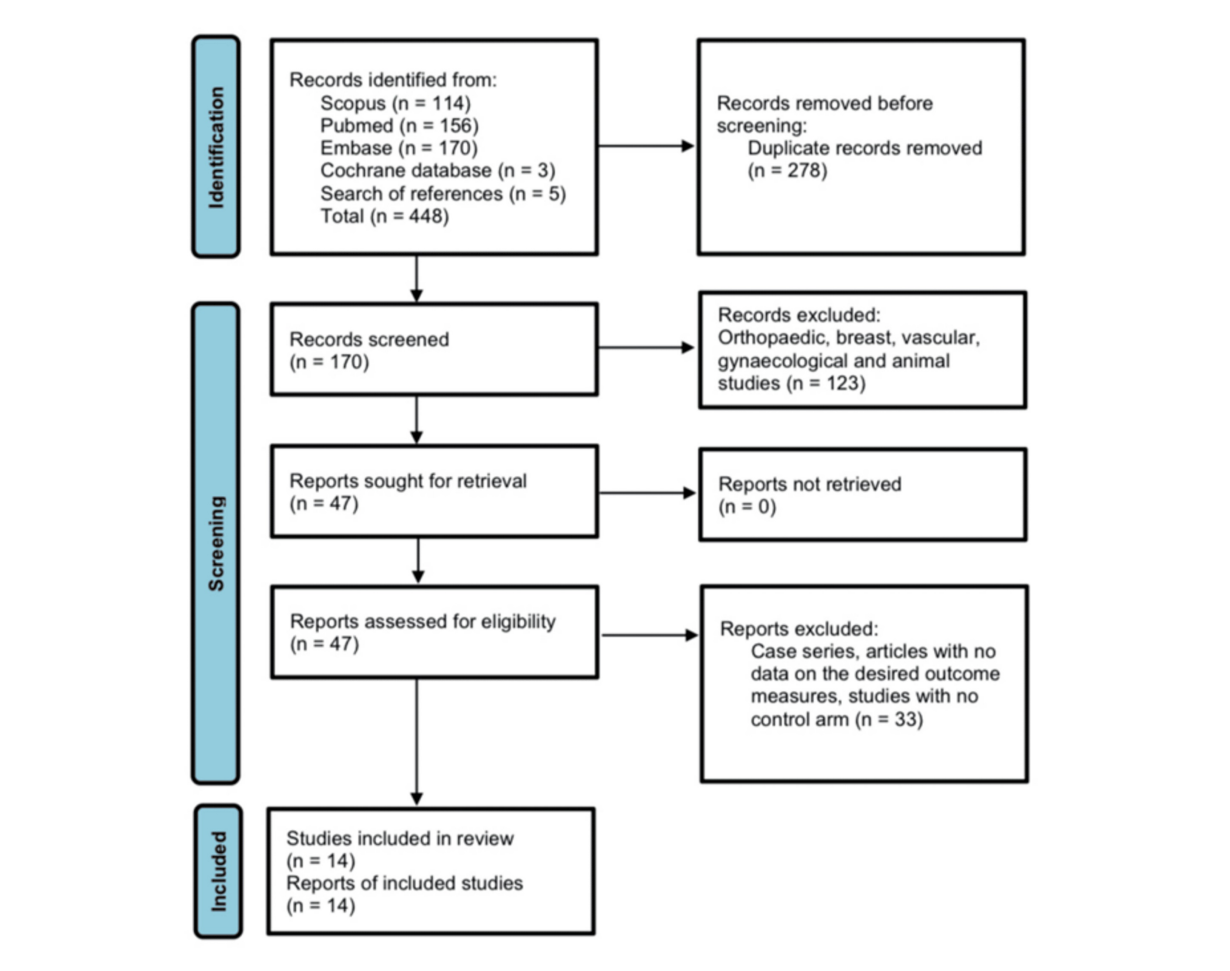
PRISMA 2020 flow diagram for updated systematic reviews, which includes searches of databases and registers PRISMA: Preferred Reporting Items for Systematic Reviews and Meta-Analyses

Search of the literature databases identified 448 articles that assessed the use of antibiotic-impregnated implants in wound management. After the exclusion of 278 duplicate studies, the remaining 170 were assessed, and those outside the field of colorectal surgery were excluded. The methodology and outcome measures of the 47 remnant articles were further screened to select the 14 RCTs that met the inclusion criteria for this systematic review. A Preferred Reporting Items for Systematic Reviews and Meta-Analyses (PRISMA) flowchart of the literature selection process is outlined in Figure 1.

Study Characteristics

A total of 14 articles comprising 2,233 patients were selected for this study. There were 1,114 patients in the GCI group and 1,119 in the control group. The articles, published between 1992 and 2014, were all RCTs. The GCI was placed in the anterior abdominal wall during wound closure, the pelvic cavity following total mesorectal excision, or the perineal wound in abdominoperineal resection and pilonidal sinus excision. The characteristics of the included studies are summarised in Table 1.

**Table 1:** Characteristics of the studies included in the analysis GCI = Gentamicin-Collagen Implant; RCT = Randomised Controlled Trial; IBD = Inflammatory Bowel Disease; AR: Anterior Resection; APR: Abdominoperineal Resection; LAR: Low Anterior Resection

Study	Year	Country	Surgical Pathology	Surgical Procedure	Wound Site	Study Design	Study Type	Mean Follow-Up
Vogel [19]	1992	Germany	Pilonidal disease	Pilonidal excision	Perineal	Primary closure and GCI vs Primary closure without GCI	RCT	1 year
Rutten [20]	1997	The Netherlands	Rectal cancer	AR, APR, Hartmann’s	Abdominal, Perineal	Closure with GCI vs Closure without GCI	RCT	-
Gomez [21]	1999	Mexico	GI perforation	Resection	Abdominal	Primary closure & GCI vs Delayed primary closure & systemic antibiotics	RCT	-
Gruessner [22]	2001	Germany	Rectal cancer	APR	Perineal	Closure with GCI vs Closure without GCI	RCT	8 weeks
Holzer [23]	2003	Germany	Pilonidal disease	Pilonidal excision	Perineal	Primary closure and GCI vs Open wound treatment	RCT	26 weeks
Nowacki [24]	2004	Poland	Rectal cancer	AR, APR, Hartmann’s	Abdominal, Perineal	Closure with GCI vs Closure without GCI	RCT	42 months
Haase [25]	2005	Germany	Loop ileostomy	Ileostomy closure	Abdominal	GCI vs Collagen alone	RCT	-
Rao [26]	2009	United Kingdom	Pilonidal disease	Pilonidal excision	Perineal	Primary closure and GCI vs Open wound treatment	RCT	5 years
Andersson [27]	2010	Sweden	Pilonidal disease	Pilonidal excision	Perineal	Primary closure and GCI vs Primary closure without GCI	RCT	1 year
Bennett-Guerrero [28]	2010	USA	Colorectal conditions	Colorectal resections	Abdominal, Perineal	Wound closure and GCI vs Wound closure alone	RCT	60 days
Yetim [29]	2010	Turkey	Pilonidal disease	Pilonidal excision	Perineal	Primary closure and GCI vs Primary closure without GCI	RCT	12 months
Collin [30]	2013	Sweden	Rectal cancer/IBD	AR, LAR	Abdominal, Perineal	Closure with GCI vs Closure without GCI	RCT	5 years
Pochhammer [31]	2014	Germany	Colorectal conditions	Colorectal resections	Abdominal	GCI vs Collagen alone vs No collagen/GCI	RCT	32 days
Rutkowski [32]	2014	Poland	Rectal cancer	AR, APR	Abdominal, Perineal	Closure with GCI vs Closure without GCI	RCT	90 days

Five studies [20,22,24,30,32] assessed the use of gentamicin-collagen implant in rectosigmoid cancer resections, 5 studies [19,23,26,27,29] on pilonidal sinus excision, 1 study [25] on loop ileostomy reversal, 1 study [21] on gastrointestinal perforations (diverticular, appendiceal and gallbladder), and 2 studies [28,31] were on surgeries for different colorectal conditions (cancer, inflammatory bowel disease, perforated diverticulitis etc).

Risk of Bias and Certainty of Evidence in the Assessed Studies

Overall, all the studies were well-controlled randomised trials and had a low risk of bias. The meta-analysis, based on randomised controlled trials, showed high-quality evidence for individual outcomes and moderate-quality evidence for the overall surgical site infection (SSI) outcome. In colorectal surgery, the overall SSI risk was 114 per 1,000 patients (11.4%) with GCI use, compared to 210 per 1,000 patients (21.0%) with placebo. The certainty of evidence for each outcome is presented in Table 2.

**Table 2:** Certainty of evidence grading of individual studies

Summary of findings						
Gentamicin-Collagen Implant Compared to Placebo in the Prevention of Surgical Site Infection in Colorectal Surgery: A Systematic Literature Review and Meta-Analysis						
Patient or population: Patients undergoing colorectal surgery Setting: Intervention: Gentamicin-Collagen Implant Comparison: Placebo						
Outcomes	Anticipated absolute effects^*^ (95% CI)		Relative effect (95% CI)	№ of participants (studies)	Certainty of the evidence (GRADE)	Comments
	Risk with Placebo	Risk with Gentamicin - Collagen Implant				
Overall Surgical Site Infection in Colorectal Surgery	210 per 1,000	114 per 1,000 (74 to 179)	RR 0.54 (0.35 to 0.85)	2070 (12 RCTs)	⨁⨁⨁◯ Moderate^a^	Gentamicin-Collagen Implant likely results in a reduction in overall surgical site infection in colorectal surgery.
Surgical Site Infection in Rectal Surgery	188 per 1,000	105 per 1,000 (73 to 152)	RR 0.56 (0.39 to 0.81)	809 (5 RCTs)	⨁⨁⨁⨁ High	Gentamicin-Collagen Implant results in a large reduction in surgical site infection in rectal surgery.
Surgical Site Infection in Pilonidal Sinus Surgery	312 per 1,000	106 per 1,000 (28 to 384)	RR 0.34 (0.09 to 1.23)	319 (3 RCTs)	⨁⨁⨁⨁ High	Gentamicin-Collagen Implant does not reduce surgical site infection in pilonidal sinus surgery.
Failure of Primary Wound Healing	286 per 1,000	203 per 1,000 (143 to 291)	RR 0.71 (0.50 to 1.02)	358 (3 RCTs)	⨁⨁⨁⨁ High	Gentamicin-Collagen Implant results in little to no difference in failure of primary wound healing.
Recurrence of Pilonidal Disease	49 per 1,000	31 per 1,000 (4 to 226)	RR 0.62 (0.09 to 4.57)	323 (4 RCTs)	⨁⨁⨁⨁ High	Gentamicin-Collagen Implant does not reduce the recurrence of pilonidal disease.
*The risk in the intervention group (and its 95% confidence interval) is based on the assumed risk in the comparison group and the relative effect of the intervention (and its 95% CI). CI: confidence interval; RR: risk ratio. Explanation a. Though all the studies were randomised controlled trials, the nature of the surgeries performed was heterogeneous and included rectal surgeries, surgeries for pilonidal disease and surgeries involving the colon and small bowel. This introduced a high degree of heterogeneity in the pooled analysis.						
GRADE Working Group grades of evidence. High certainty: We are very confident that the true effect lies close to that of the estimate of the effect. Moderate certainty: We are moderately confident in the effect estimate: the true effect is likely to be close to the estimate of the effect, but there is a possibility that it is substantially different. Low certainty: Our confidence in the effect estimate is limited; the true effect may be substantially different from the estimate of the effect. Very low certainty: we have very little confidence in the effect estimate; the true effect is likely to be substantially different from the estimate of the effect.						

Results of Individual Studies

Out of the 14 studies, only 2 did not consider surgical site infection as the primary outcome measure [23,26]. Three studies assessed the proportion of patients who had primary wound healing [22,27,30]. In terms of disease recurrence, four studies assessed the recurrence of pilonidal disease [19,23,26,29]. Data on the selected outcomes of the individual studies is summarised in Table 3.

**Table 3:** Results of primary and secondary outcomes in the studies used for the analysis GCI = Gentamicin-Collagen Implant; values in brackets indicate the number of patients that had failed primary wound healing

Study	Total		Surgical Site Infection		Primary Wound Healing		Disease Recurrence	
	GCI	Control	GCI	Control	GCI	Control	GCI	Control
Vogel 1992 [19]	40	40	3	21	-	-	0	0
Rutten 1997 [20]	107	114	6	21	-	-	-	-
Gomez 1999 [21]	34	32	3	14	-	-	-	-
Gruessner 2001 [22]	49	48	3	10	47 (2)	44 (4)	-	-
Holzer 2003 [23]	51	52	-	-	-	-	1	0
Nowacki 2004 [24]	106	112	6	10	-	-		
Haase 2005 [25]	40	40	4	4	-	-	-	-
Rao 2009 [26]	30	30	-	-	-	-	2	2
Andersson 2010 [27]	82	77	18	20	63 (19)	51 (26)	-	-
Bennett-Guerrero 2010 [28]	300	302	90	63	-	-	-	-
Yetim 2010 [29]	40	40	2	8	-	-	0	6
Collin 2013 [30]	52	50	10	14	36 (16)	30 (20)		
Pochhammer 2014 [31]	97	97	8	11	-	-	-	-
Rutkowski 2014 [32]	86	85	16	22	-	-	-	-
Total	1114	1119	169	218	146 (37)	125 (50)	3	8

Results of the Pooled Analysis

**Figure 2:**
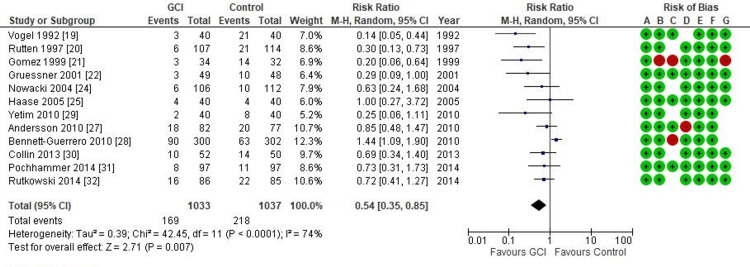
Forest plot of comparison: Gentamicin-Collagen Implant (GCI) versus control Outcome: Overall surgical site infection

Surgical site infection: A pooled random-effect meta-analysis of the data showed that the overall SSI rate was significantly lower in the GCI group than in the control group (RR = 0.54; CI = 0.35 - 0.85; p = 0.007). As illustrated in Figure 2, there was significant heterogeneity among the studies.

**Figure 3:**
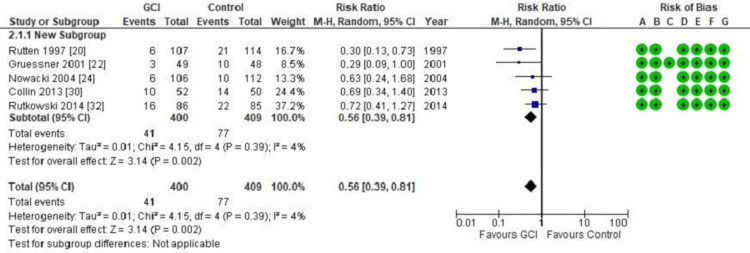
Forest plot of comparison: Gentamicin-Collagen Implant (GCI) versus control Outcome: Surgical site infection following rectal cancer surgeries

A pooled subgroup analysis of SSI among patients who had radical resections (anterior resection, abdominoperineal resection, Hartmann’s procedure) for rectal cancer showed that the use of GCI significantly decreased the rate of infection of the abdominal or perineal surgical wound (p = 0.002). As shown in the forest plot in Figure 3, the random effect RR was 0.56 and the 95% confidence interval was 0.39-0.81, with a low heterogeneity of 4%.

**Figure 4:**
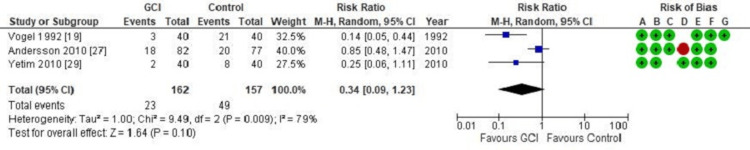
Forest plot of comparison: Gentamicin-Collagen Implant (GCI) versus control Outcome: Surgical site infection following excision and primary closure of pilonidal sinus

A further subgroup analysis of surgeries performed for pilonidal disease revealed no significant difference in the SSI rate between the GCI group and the control (RR = 0.34; CI = 0.09 - 1.23; p = 0.10), as shown in Figure 4.

**Figure 5:**
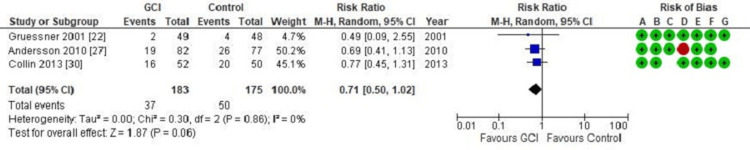
Forest plot of comparison: Gentamicin-Collagen Implant (GCI) versus control Outcome: Failure of primary wound healing

Primary wound healing: Figure 5 is a forest plot that illustrates the incidence of failure of primary wound healing, utilising 183 patients in the GCI group and 175 patients in the control group. There is no statistically significant difference in the rate of primary wound healing failure between the GCI group and those without GCI (RR = 0.71; CI = 0.50 - 1.02; p = 0.06).

**Figure 6:**
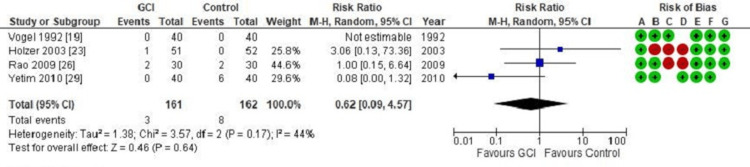
Forest plot of comparison: Gentamicin-Collagen Implant (GCI) versus control Outcome: Recurrence of pilonidal disease

Disease recurrence: Figure 6 is a forest plot illustrating the pooled analysis of recurrence of pilonidal disease following excision and primary closure. There were 161 patients in the intervention group and 162 patients in the control group. The use of GCI had no significant impact on the reduction of pilonidal disease recurrence (RR = 0.62; CI = 0.09 - 4.57; p = 0.64).

Discussion

Many RCTs have been conducted to evaluate the effectiveness of GCI in the prevention of SSI in colorectal surgery, but the results of these individual studies have been conflicting. While Vogel et al., Rutten et al. and Gomez et al. reported a statistically significant reduction in the incidence of SSI with the use of GCI, Haase et al. and Pochhammer et al. observed no significant difference [19-21,25,31]. In fact, Bennett-Guerrero and colleagues reported an increased incidence of SSI among patients who had GCI during their wound closure [28]. These inconsistent results prompted the undertaking of the current study, which seeks to integrate the results of relevant studies and provide a clear, concise summary that clinicians can readily apply in practice.

Wang and colleagues, in their 2023 article, performed a meta-analysis to assess the effectiveness of prophylactic topical antimicrobials in the prevention of surgical wound infections in colorectal surgery. In this study, topical antimicrobials significantly reduced the incidence of surgical wound infections (p < 0.001) compared to controls. However, the data used for the meta-analysis were obtained from heterogeneous studies that utilised a variety of different topical antimicrobials, including gentamicin-collagen sponge and beads, triclosan-coated fascial sutures, ionised silver dressing on closed abdominal wounds, and antibiotic powder, ointment, lavage or irrigation for the abdominal wound [33].

Our study reviewed a total of 14 studies consisting of 2,233 patients: 1,114 with and 1,119 without GCI. A meta-analysis of the pooled data found a significant reduction in the overall SSI incidence with the use of GCI (RR = 0.54; CI = 0.35 - 0.85; p = 0.007). The overall SSI risk was 114 per 1,000 patients (11.4%) with GCI use, compared to 210 per 1,000 patients (21.0%) with placebo.

This finding is also similar to the results of the meta-analysis on SSI conducted by Chang and co-workers. In their study, which included surgeries in other anatomical regions outside the colorectal area, the overall SSI rate was significantly reduced by the use of GCI (p = 0.001). This difference was also maintained in a subgroup analysis for clean (p = 0.01) and clean-contaminated (p = 0.03) surgeries [8].

We performed a subgroup analysis on the SSI incidence among patients who had GCI for rectal cancer surgeries, as well as those who had excision and primary closure of pilonidal sinus. In the former subgroup, GCI application in the pelvic cavity (after excision of the rectum) and in the perineal wound (following abdominoperineal resection) significantly reduced the incidence of SSI in the wounds (RR = 0.56, CI = 0.39 - 0.81, p = 0.002). Among the subgroup of patients with pilonidal disease, however, GCI did not reduce the SSI rate (RR = 0.34; CI = 0.09 - 1.23; p = 0.10). This result, observed among patients with pilonidal disease, may have been affected by the small sample size, as only three studies (with a total of 319 patients) were eligible for the analysis.

A meta-analysis of the primary wound healing rate with and without the use of GCI revealed that patients who had GCI during wound closure did not achieve better primary wound healing (or less wound healing failure) than those without GCI. This indicates that the incidence of postoperative wound complications that prevent or delay primary wound healing is not affected by the use of GCI (RR = 0.71; CI = 0.50 - 1.02; p = 0.06). This contrasts with the results obtained following cardiac surgery, in which GCI significantly improved the wound healing rate [7,34].

An analysis of disease recurrence revealed no significant difference in the recurrence rate of pilonidal disease after previous excision, whether GCI was used or not (RR = 0.62; CI = 0.09 - 4.57; p = 0.64). This finding could be related to the failure of GCI to reduce the rate of SSI in pilonidal surgery.

Limitations

The limitations of this study include high heterogeneity obtained in the analysis of the overall SSI. This may be due to the disparate locations of the surgical wounds (abdominal and perineal), the differences in surgical pathologies, procedures performed and anatomical sites where the gentamicin-collagen implants were placed. This was mitigated by subgroup analysis of the SSI based on the site of surgery. Despite the significant heterogeneity, however, the overall risk of bias of the study is very low, as all the component studies were RCTs.

Over the past three decades, surgical techniques have evolved significantly -- from open procedures to laparoscopic and later robotic approaches -- leading to notable changes in the risk profile for SSIs in colorectal surgery. As many of the studies included in this meta-analysis were conducted using the open surgical technique, the findings may not fully reflect outcomes associated with minimally invasive approaches.

Variations across studies in the implementation of effect modifiers, such as the use of the ERAS protocol, anatomical wound site, neoadjuvant therapy, collagen brand or dosage and different perioperative antibiotic protocols, may have influenced the study outcomes. To minimise the impact of these confounding variables, a subgroup meta-analysis was conducted.

## Conclusions

Surgical site infection is a common cause of postoperative morbidity following colorectal surgeries. Gentamicin-containing collagen sponges deliver a high concentration of gentamicin within the local surgical site. Slow release of the drug for a longer period in the surgical site reduces systemic absorption, risk of antibacterial drug resistance and the need for repeated systemic antibiotic administration.

In this systematic review and meta-analysis, the use of a GCI significantly reduced the incidence of SSI in colorectal surgery and, specifically, in rectal surgeries. In colorectal surgery, the overall SSI risk was 114 per 1,000 patients (11.4%) with GCI use, compared to 210 per 1,000 patients (21.0%) with placebo. GCI has not been shown to reduce the incidence of SSI or disease recurrence following surgeries for pilonidal disease.
